# Diagnostic test accuracy of loop-mediated isothermal amplification assay for Mycobacterium tuberculosis: systematic review and meta-analysis

**DOI:** 10.1038/srep39090

**Published:** 2016-12-13

**Authors:** Kenjiro Nagai, Nobuyuki Horita, Masaki Yamamoto, Toshinori Tsukahara, Hideyuki Nagakura, Ken Tashiro, Yuji Shibata, Hiroki Watanabe, Kentaro Nakashima, Ryota Ushio, Misako Ikeda, Atsuya Narita, Akinori Kanai, Takashi Sato, Takeshi Kaneko

**Affiliations:** 1 Department of Pulmonology, Yokohama City University Graduate School of Medicine, 3–9, Fukuura, Kanazawa, Yokohama, Japan

## Abstract

Diagnostic test accuracy of the loop-mediated isothermal amplification (LAMP) assay for culture proven tuberculosis is unclear. We searched electronic databases for both cohort and case-control studies that provided data to calculate sensitivity and specificity. The index test was any LAMP assay including both commercialized kits and in-house assays. Culture-proven M. tuberculosis was considered a positive reference test. We included 26 studies on 9330 sputum samples and one study on 315 extra-pulmonary specimens. For sputum samples, 26 studies yielded the summary estimates of sensitivity of 89.6% (95% CI 85.6–92.6%), specificity of 94.0% (95% CI 91.0–96.1%), and a diagnostic odds ratio of 145 (95% CI 93–226). Nine studies focusing on Loopamp MTBC yielded the summary estimates of sensitivity of 80.9% (95% CI 76.0–85.1%) and specificity of 96.5% (95% CI 94.7–97.7%). Loopamp MTBC had higher sensitivity and lower specificity for smear-positive sputa compared to smear-negative sputa. In-house assays showed higher sensitivity and lower specificity compared to Loopamp MTBC. LAMP promises to be a useful test for the diagnosis of TB, however there is still need to improve the assay to make it simpler, cheaper and more efficient to make it competitive against other PCR methods already available.

*Mycobacterium (M.) tuberculosis* (TB) is a life threatening infectious disease affecting both the HIV-infected and HIV-non-infected population. TB frequently affects human lungs and causes a variety of symptoms such as fatigue, wet cough, bloody sputum, and persistent fever. Although the worldwide incidence and prevalence of TB are gradually decreasing, approximately 1.5 million deaths a year are attributed to TB according to the World Bank and the World Health Organization^1^. It is indispensable to obtain accurate TB diagnosis to treat and to prevent the spread of TB. Acid-fast stain and culture are classical examinations for TB. However, their diagnostic test accuracy, especially sensitivity for smear, is not sufficient. Accurate TB diagnosis by culture requires long incubation time^2^,^3^.

Since being developed in 1983, the polymerase chain reaction (PCR) has played a central role in nucleic acid amplification. Currently, some PCR kits such as Cobas TaqMan and Xpert MTB/RIF are commercially available and are widely used for TB diagnosis^4^,^5^. However, the PCR assay requires an expensive thermal cycler to amplify the DNA fragment in multiple temperature-dependent steps. The loop-mediated isothermal amplification (LAMP) assay is another nucleic acid amplification technique. In contrast to the PCR, the LAMP assay can amplify a targeted sequence at a constant temperature. Therefore, a large and costly thermal cycler is not necessary for a LAMP assay^6^,^7^. An inexpensive LAMP would be especially welcomed in such area which have a shortage of medical equipment. Brazil, Russia, east Asian, south Asian, south-east Asian, south African, and east African countries have a high-burden of tuberculosis. Medical resources are limited in most of these countries^1^. Availability of expertise and technology in the peripheral area is always a considerable issue. Quality of any form of diagnosis is often even worse in peripheral hospitals of developing countries.

During the last 10 years, several researchers have assessed the diagnostic test accuracy of the LAMP assay for tuberculosis. Although these studies have revealed generally very good diagnostic performance, there are considerable discrepancies between their results^8^,^9^,^10^,^11^,^12^,^13^,^14^,^15^,^16^,^17^,^18^,^19^,^20^,^21^,^22^,^23^,^24^,^25^,^26^,^27^,^28^,^29^,^30^,^31^,^32^. In addition, none of the studies could describe precise diagnostic accuracy because of their limited statistical power. Two research groups conducted systematic reviews and univariate meta-analyses to estimate the pooled sensitivity and specificity^6^,^7^. However, we believe an updated meta-analysis is required. This is because these systematic reviews indicated discrepant results, i.e. the pooled sensitivity of 80% and 93%, and because recent meta-analysis guidelines have strongly suggested a hierarchical meta-analysis approach instead of simple univariate meta-analysis^33^,^34^. The aim of the current systematic review and meta-analysis is to reveal the diagnostic test accuracy of the LAMP assay for tuberculosis using the data from previous studies.

## Methods

### Study registration

The study protocol followed the Cochrane Handbook for Diagnostic Test Accuracy Reviews and the Preferred Reporting Items in Systematic Reviews and Meta-Analyses statement^34^,^35^. This protocol has been registered with the international prospective register of systematic reviews (PROSPERO) as number CRD42016032722^36^.

### Eligibility criteria

#### Type of studies

We included both cohort and case-control studies. A study for diagnostic test accuracy is essentially cross-sectional. However, single- and two-gate studies are customarily termed cohort and case-control studies in this field^33^. We considered that case-control studies had a high risk of bias for patient selection^37^. Even though an article did not directly provide the diagnostic accuracy of the LAMP assay for TB, if it had sufficient data to calculate the sensitivity and the specificity, it was included.

Following the protocol, we did not exclude studies only because of non-English description. Our protocol also allowed non-full articles such as conference reports.

#### Index and reference test

We considered any LAMP assays targeting TB nucleic acid including both commercialized kits and in-house assays as index tests. Specimens should be clinical specimens and culture isolates were not accepted.

We used culture-proven *M. tuberculosis* as a reference test. In addition to *M. tuberculosis*, other species belonging to the *M. tuberculosis complex*, such as *M. bovis* and *M. africanum*, were also regarded as *M. tuberculosis* because in normal clinical situations, it is practically impossible to distinguish them from *M. tuberculosis*^2^.

### Literature search strategy

On December 28th, 2015, we searched Pubmed, EMBASE, the Cochrane Library on Wiley, and Web of Science.

We used the following formula for Pubmed without any limitation: (Tuberculosis OR TB OR mycobacteri*) AND (LAMP OR “loop-mediated isothermal amplification”) AND (sensitivity OR specificity OR “predictive value” OR likelihood OR “true positive” OR “true negative” OR “false positive” OR “false negative”). Similar search formulas were also used for Embase, Cochrane library, and Web of Science (Supplementary Text 1).

References of previously published reviews and those of included original studies were checked for possible candidate articles.

### Study screening and selection

Two investigators (KN, NH) independently screened candidate articles by checking the title and abstract. Once independent screening was finished, articles that were regarded as candidates by at least one investigator were examined by the two investigators for final inclusion. Discrepancies were resolved by discussion between the two investigators.

### Data extraction

The two investigators independently extracted necessary information from the finally included articles Then, we cross-checked the data extracted by the two investigators. Discrepancies were resolved by discussion between the two investigators.

Respiratory specimens and non-respiratory specimens were treated separately. When respiratory and non-respiratory specimens were evaluated collectively in a study and we could not separate these data, we disregarded the data.

### Quality assessment for bias and applicability

The two investigators independently assessed the seven domains of a Revised Tool for the Quality Assessment of Diagnostic Accuracy Studies (QUADAS-2)^37^. A study that had no domain with a high risk of bias and no domain with high applicability concerns was regarded as a high-quality study.

### Statistical analysis

#### Outcomes and data synthesis

First, we made two by two contingencies from the number of true positives/false negatives/false positives/true negatives presented in each original study. These numbers were counted based on specimens, not persons. To assess the overall accuracy, we evaluated the diagnostic odds ratio (DOR) using the DerSimonian-Laird random-model and the area under hierarchical summary receiver operating characteristics (HSROC) curves (AUC) using Holling’s proportional hazard model^38^,^39^. We also drew a paired forest plot and HSROC, and calculated the summary estimates of the sensitivity and the specificity using the bivariate model^33^. The positive likelihood ratio (PLR) and negative likelihood ratio (NLR) were estimated based on the summary estimates of sensitivity and specificity^33^,^34^. AUC, PLR, and NLR were interpreted according to the criterion by Grimes *et al*. and Jones *et al*.^40^,^41^.

#### Heterogeneity

The heterogeneity assessed by the I^2^ statistic was interpreted as follows: 0% to 40% was not be important, 30% to 60% represented moderate heterogeneity, 50% to 90% represented substantial heterogeneity, 75% to 100% indicated considerable heterogeneity^42^.

#### Software

We used the following commands of the statistics software R: the “madauni” command for DOR, the “phm” command for AUC, and the “reitsma” command for the HSROC curve and the summary estimates of sensitivity and specificity^38^,^39^.

#### Sensitivity analysis

We conducted sensitivity analyses by focusing on high-quality reports, the in-house LAMP assay, and by using the commercialized Loopamp MTBC Detection Kit targeting gyrB DNA and IS6110 DNA manufactured by EIKEN CHEMICAL CO., LTD. (Loopamp MTBC). Use of only the Loopamp PURE DNA Extraction Kit was not counted as Loopamp MTBC. We also evaluated the diagnostic test accuracy using data derived from studies that obtained one sputum specimen from an individual.

## Results

### Study search and study characteristics

**Figure 1:**
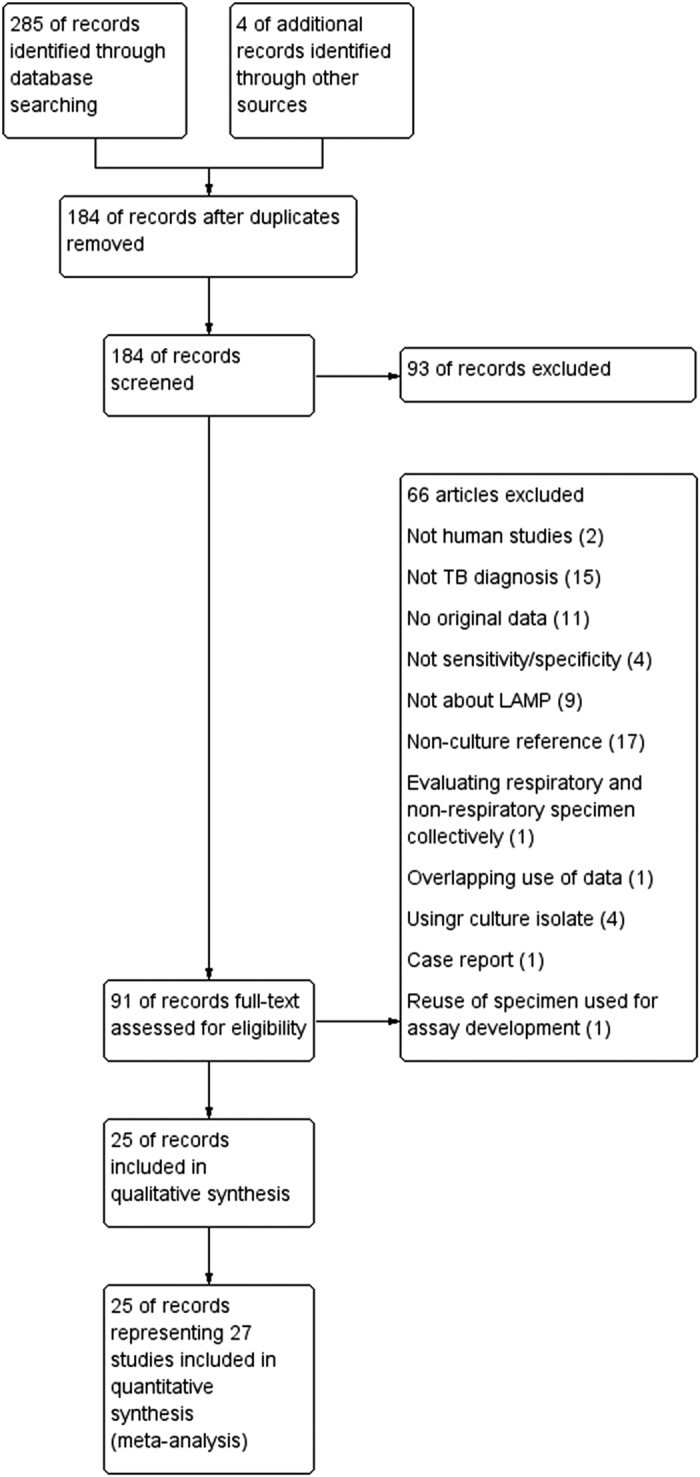
The study search flow chart. We found 70, 72, 133, and 10 articles from Pubmed, Web of Science, EMBASE, and Cochrane database, respectively.

Of the 289 candidate articles, we finally identified 25 eligible articles representing 27 independent studies (Fig. 1).

Among the 27 studies, 22 used the cohort approach, one used the case-control approach, and four did not describe the recruitment approach; 17 were full-length articles, nine were conference reports, one was thesis; 24 were written in the English language, three were written in the Japanese language; nine used the commercialized Loopamp MTBC, 18 evaluated in-house LAMP assays; 26 evaluated sputum samples, and one evaluated extra-pulmonary specimens (Table 1). No study used non-sputum respiratory samples such as gastric fluid or bronchial lavage. Notably, a conference report by the World Health Organization (WHO) described three independent studies^11^. While, three were multi-national studies, 24 were single-national studies. Six were from each of Japan, and India, three were from each of China and Iran, and one was from each of five other countries. According to the World Bank classification, eight, seven, eight, and one were from high, upper-middle, lower-middle, and low income countries, respectively. The number of specimens evaluated in each study ranged from 10 to 1741 with a median of 161, which totaled 9645 consisting of 3099 TB culture-positive specimens and 6546 TB culture negative specimens. Six studies were regarded as low-quality due to the case-control study design or inappropriate exclusion of samples, while the other 21 were regarded as high-quality studies (Table 1. Supplementary Figure 1).

### Respiratory specimen

**Figure 2:**
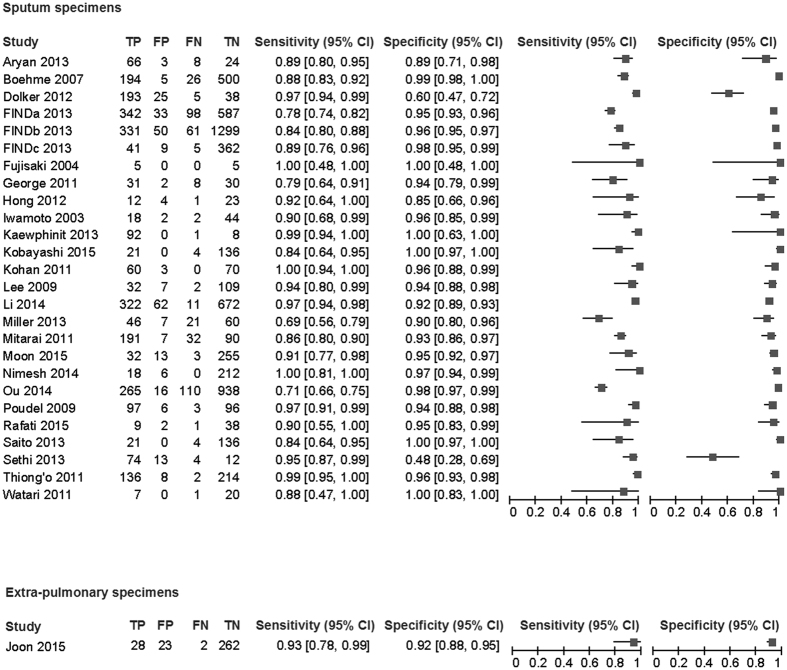
The paired forest plots.

**Figure 3:**
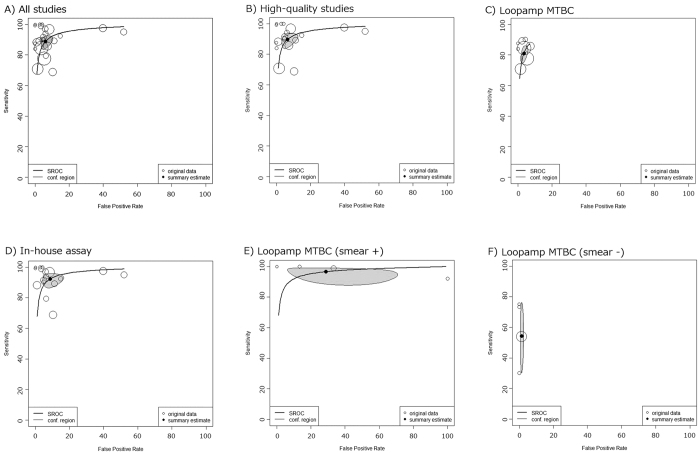
Hierarchical summary receiver operating characteristics curves for studies evaluating sputum samples. Size of circles indicates weight of each study.

In the 26 studies that evaluated 9330 sputum samples included 3069 culture positive specimens and 6261 culture negative specimens were assessed. These studies yielded sensitivities in the range of 68.7–100.0% with a median of 90.0% and specificities in the range of 48.0–100.0% with a median of 95.4% (Fig. 2). The AUC of 0.962 (95% confidence interval (95% CI) 0.949–0.975) and the DOR of 145 (95% CI 93–226, I^2^ = 19.8%) suggested very good overall diagnostic accuracy (Table 2, Figs 2 and 3A)^41^. The data from the 26 studies provided the summary estimate sensitivity of 89.6% (95% CI 85.6–92.6%) and the summary estimate specificity of 94.0% (95% CI 91.0–96.1%). PLR and NLR were 14.9 (95% CI 9.8–22.8) and 0.11 (95% CI 0.08–0.15), respectively. These likelihood ratios meant that a positive LAMP assay result greatly increased the probability of culture proven TB and that a negative LAMP assay result moderately decreased the probability^40^.

After six low-quality studies were excluded, the diagnostic accuracy statistics did not significantly change (Table 2, Fig. 3B).

Based on assay-dependent subgroup analyses, Loopamp MTBC was evaluated by nine studies on 5283 sputum samples and the in-house LAMP assay was evaluated by 17 studies on 4047 sputum samples(Table 2, Fig. 3C,D). Although the in-house assays used a variety of analysis methods and targeted nucleic acids, we did not observe heterogeneity during the meta-analysis of DOR for in-house assays (I^2^ = 0%). There was no significant DOR value difference between studies focusing on Loopamp MTBC and studies focusing on in-house LAMP. However, the AUC was significantly higher for studies focusing on in-house assays. The summary estimate of sensitivity for in-house assays (sensitivity 93.0%, 95% CI 88.9–95.7%) was higher than that for Loopamp MTBC (sensitivity 80.9%, 95% CI 76.0–85.1%), while the summary estimate of specificity for in-house assays (specificity 91.8%, 95% CI 86.4–95.1%) was lower than that for Loopamp MTBC (specificity 96.5%, 95% CI 94.7–97.7%).

**Figure 4:**
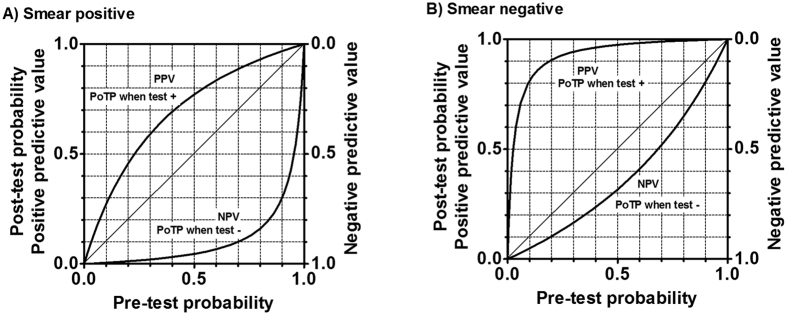
Predictive values of the Loopamp MTBC assay depending on sputum smear status and pre-test probability. PPV: positive predictive value. NPV: negative predictive value. PoTP: post-test predictive value. The figure was drawn based on the sensitivity of 0.966 and specificity of 0.713 for smear-positive sputum samples and the sensitivity of 0.543 and specificity of 0.986 for smear-negative sputum samples. Pre-test probability has similar meaning to prevalence. Readers can simply input the prevalence in the country or area into pre-test probability. For example, in the area of prevalence of 5%, the pre-test probability may be 5% for screening setting. However, if clinical information is available, patient specific pre-test probability is preferred. For example, the pre-test probability may be 50% for a patients with chronic fever, history of TB contact, and a cavitation on X-ray even in an area with prevalence of 5%.

Four studies provided data concerning the diagnostic test accuracy of Loopamp MTBC for smear-positive and negative specimens separately. For smear-positive sputa, the Eiken assay yielded the summary estimate sensitivity of 96.6% (95% CI 90.4–98.8%) and specificity of 71.3% (95% CI 37.1–91.3%). These summary estimates resulted in PLR of 3.4 (95% CI 1.5–11.1) and NLR of 0.05 (95% CI 0.02–0.16) (Table 2, Fig. 3E). For smear-negative sputa, the Eiken assay led to the summary estimate sensitivity of 54.3% (95% CI 34.7–72.6%) and specificity of 98.6% (95% CI 97.3–99.1%), which yielded PLR of 38.8 (95% CI 19.3–72.5) and NLR of 0.46 (95% CI 0.28–0.66) (Table 2 Fig. 3F). PPVs and NPVs depending on the pre-test probability and smear status were estimated from these summary estimates of sensitivity and specificity (Fig. 4).

As a sensitivity analysis, we evaluated the diagnostic test accuracy using data derived from studies that obtained a sputum specimen from an individual. This provided DOR of 130 (95% CI 73–231) and AUC of 0.958 (95% CI 0.939–0.979), which are compatible those obtained from the data of 26 studies with sputum (Table 2G).

### Extra-pulmonary specimen

Only one study by Joon *et al*. evaluated the diagnostic test accuracy of the Loopamp MTBC assay for culture-proven TB using extra-pulmonary specimens. This study assessed 315 extra-pulmonary specimens including blood, urine, lymph node, and a variety of body fluids (Fig. 2). They revealed a DOR of 159 (95% CI 36–712), the AUC of 0.961, a summary estimate sensitivity of 93.3% (95% CI 77.9–99.2%), and a summary estimate of specificity of 91.9% (95% CI 88.1–94.8%) (Table 2).

## Discussion

We systematically reviewed the diagnostic accuracy of the LAMP assay for culture proven TB. Judging from the DOR and the AUC, the diagnostic accuracy of LAMP assay was very good for both sputum and non-respiratory specimens^41^. However, its diagnostic test accuracy was poorer than that of PCR assays such as Xpert and Cobas TaqMan^4^,^5^,^43^. Using sputum samples, the LAMP assay provided the summary estimate sensitivity of 89.6% and specificity of 94.0%. We did not observe any significant discrepancy between test accuracies between sputa and extra-pulmonary specimens (Table 2). The robustness of our result was supported by numerous factors. First, we carried out an attentive study search. Yuan *et al*. reported a systematic review on the same topic in 2014, which finally included 10 articles with 1920 specimens^6^. A recently published systematic review by Yan *et al*. included nine articles with 2971 sputum samples^7^. In contrast, we were able to include as many as 26 studies with 9285 specimens thanks to our careful study searching. This large number of included studies and specimens enhanced the credibility of our analysis. Second, across our meta-analyses, we did not find strong heterogeneity (I^2^ < 30%). Third, sensitivity analyses consistently revealed similar overall diagnostic ability. Fourth, the currently recommended hierarchical model was used instead of univariate meta-analysis. In addition, the strength of the current study is that we obtained diagnostic accuracy focusing on Loopamp MTBC and smear statuses.

Nucleic acid amplification shows different diagnostic characteristics depending on the smear status (Table 2, Figs 3 and 4)^5^. Thus, we have to interpret the result from the LAMP assay combined with the result from smear status. For smear-negative specimens, a positive LAMP assay greatly increased the probability of culture-proven TB and can be recognized as a rule-in examination. However, a negative LAMP assay could not add essential information on smear-negative specimens. A PCR assay also frequently shows a false negative result for smear-negative sputa, thus the diagnostic test accuracy of the LAMP assay for smear-negative sputa is almost comparable to that of PCR^5^. For smear-positive specimens, a negative LAMP assay greatly decreased the probability of TB. However, because of the frequent false positive LAMP assay results for smear-positive sputa, a positive LAMP for smear-positive sputa did not greatly affect the probability of TB. High temperature, humidity, and inadequate volume of reagents are known risk factors for false positive results. This assay produces a large amount of DNA, which often spreads into the open air. This may cause crossover contamination in the subsequent assay. In addition, Loopamp MTBC may also cross-react with human DNA^11^. To minimize the risk of false positives, comprehensive training of laboratory technicians is required.

Loopamp MTBC, which is the only commercialized LAMP kit for TB, is not endorsed by WHO^11^. Among 27 included studies, only nine evaluated the Loopamp MTBC and the other 18 evaluated in-house LAMP assays. It is not very difficult to design original LAMP primers, thus researchers often try to create their original LAMP assay. Compared to Loopamp MTBC, the in-house LAMP assay showed higher sensitivity and lower specificity (Table 2, Fig. 3). However, high performance of in-house assay is potentially supported by the bias introduced by higher skill and abundant resources in qualified laboratories that can conduct original LAMP assay compared to peripheral laboratories that participated in many field researches for the commercialized kit. The TB LAMP assay is usually applied for TB-suspected patients and is rarely used for screening purpose. To rule-in the TB diagnosis, specificity is more important than sensitivity. Therefore, reported in-house assays are generally not attractive. When designing a novel LAMP assay for TB, an assay with high specificity will be preferred.

There are variety of examinations to detect TB, thus physicians have to select the optimal diagnostic strategy according to the pre-test probability of TB estimated from patient characteristics and the prevalence of TB in the area. In addition, budget issues concerning both the patients and local government should be considered. Although PCR assays are the best examinations currently available^11^, LAMP is being accepted as an alternative test in resource limited countries. In our previous systematic review concerning Cobas TaqMan MTB real-time PCR, 13 out of 17 studies were reported from high-income countries using the World Bank criteria^5^. In contrast, only nine out of 27 studies in the current systematic review for the LAMP assay were from high-income countries (Table 1). Currently, WHO recommend the use of Xpert MTB/RIF PCR assay to check the rifampicin resistance even in the peripheral laboratories^11^,^43^. Although we generally understand the advantage of Xpert MTB/RIF over LAMP, many peripheral laboratories in high TB endemic area with limited infrastructures and medical resources cannot afford Xpert MTB/RIF^44^. LAMP might replace Xpert MTB/RIF in low-income peripheral area where multi-drug resistant TB is not prevalent, though clinicians should carefully consider the result of sputum smear and epidemiology, especially concerning drug resistance, in the area^5^,^45^. Currently, neither LAMP nor Xpert is the ideal tool for peripheral setting.

Our study has a few limitations. First, some of the included studies had a risk of bias due to their study design. However, even after excluding the high-risk studies, the estimated test accuracy did not change. Second, studies about in-house assays evaluated a variety of assays. Nonetheless, meta-analysis concerning in-house assays did not show heterogeneity. We believe these flaws do not impair the robustness of our analysis. Third, our analysis does not directly answer the diagnostic test accuracy of LAMP assay for some specific settings, namely HIV-positive patients, drug-resistant TB, child cases, and non-TB mycobacterium suspected cases. Fourth, some included studies used multiple samples from one individual. Although this matter may bias the analysis result, the sensitivity analysis focusing on studies that evaluated one specimen from one patient guaranteed our overall analysis (Table 2). Lastly, some think that our study search method was not sensitive enough, though we believe the search strategy is balancing sensitivity and specificity well.

In conclusion, we conducted a systematic review and meta-analysis using solid methodology to reveal the precise diagnostic test accuracy of the LAMP assay for TB. We included nearly three times as many studies as previous systematic reviews. A sensitivity analysis ensured that our results were robust. Although the diagnostic test accuracy of LAMP assay is very good, it is still poorer than that of PCR assays. Compared to Loopamp MTBC, the in-house LAMP showed higher sensitivity and lower specificity. Diagnostic characteristics were very different, depending on smear status. We believe use of the LAMP assay combined with smear status is an acceptable diagnostic strategy especially in resource-limited areas. However there is still need to improve the assay to make it simpler, cheaper and more efficient to make it competitive against other PCR methods already available.

## Additional Information

**How to cite this article**: Nagai, K. *et al*. Diagnostic test accuracy of loop-mediated isothermal amplification assay for Mycobacterium tuberculosis: systematic review and meta-analysis. *Sci. Rep.* **6**, 39090; doi: 10.1038/srep39090 (2016).

**Publisher's note:** Springer Nature remains neutral with regard to jurisdictional claims in published maps and institutional affiliations.

## Supplementary Material

Supplementary Information

## Figures and Tables

**Table 1:** Characteristics of included studies.

Study	Country (income class)	Design	Facility	Decontamination	Stain	Culture	LAMP assay (targeted nucleic acid)	Specimen	Culture+/total	High quality
Aryan^8^	Iran (B)	pCohort	A university hospital	NALC-NaOH	ZN	LJ	In-house (IS6110)	Sp	74/101	Yes
Boehme^9^	Peru (B), Bangladesh (C), Tanzania(D)		Centers	1.5%NALC-NaOH	ZN	LJ	In-house (gyrB)	Sp	220/725	Yes
Dolker^10^	India (C)	Cohort	A Tb hospital		ZN	LJ	In-house (IS6110)	Sp	198/261	Yes
FINDa^11^	Peru (B), South Africa (B), Vietnam (C), Brazil (B)	Cohort, CR, #	DOT centers, TB clinics, a tertiary hospital	NaLC-NaOH		MGIT	Loopamp MTBC	Sp	440/1060	No
FINDb^11^	India (C), Uganda, Peru (B)	Cohort, CR, #	Hospitals, TB labs, microscopic centers	NaLC-NaOH		MGIT, LJ	Loopamp MTBC	Sp	392/1741	No
FINDc^11^	India (C)	pCohort, CR, #	Clinics	NaLC-NaOH		MGIT, LJ	Loopamp MTBC	Sp	46/417	No
Fujisaki^12^	Japan (A)	pCohort, Jpn, #	A university hospital	NALC-NaOH		Ogawa	In-house (16S rRNA)	Sp	5/10	Yes
George^13^	India (C)	pCohort	A college hospital	NALC-NaOH	AR	LJ, MGIT	In-house (rimM)	Sp	39/71	No
Hong^14^	China (B)	Cohort, #	A Tb hospital	NALC-NaOH			In-house (esat6, mtp40)	Sp	13/40	Yes
Iwamoto^15^	Japan (A)	Cohort	Community hospitals	NALC-NaOH			Loopamp MTBC	Sp	20/66	Yes
Joon^16^	India (C)	Cohort, #	A laboratory		ZN	MGIT	In-house (sdaA)	EP	30/315	Yes
Kaewphinit^17^	Thailand (B)	Cohort	A Tb laboratory	NALC-NaOH		LJ	In-house (IS6100)	Sp	93/101	Yes
Kobayashi^18^	Japan (A)	Cohort, Jpn, #	A 2ndary referral hospital	NALC-NaOH	AR	Bact/ALERT, Ogawa, Kudo	Loopamp MTBC	Sp	25/161	Yes
Kohan^19^	Iran (B)	Cohort	A Tb center	4%NaOh	ZN	LJ	In-house (IS6110)	Sp	60/133	Yes
Lee^20^	Taiwan (A)	Cohort	A university hospital	NaOH			In-house (16S rDNA)	Sp	34/150	Yes
Li^21^	China (B)	Cohort	A Tb Cenber	4%NaOH	ZN	LJ	In-house real-time (IS6011)	Sp	333/1067	Yes
Miller^22^	Zambia (C)	pCohort, CR	A chest clinic	NALC-NaOH		MGIT	In-house	Sp	67/134	Yes
Mitarai^23^	Japan (A)	pCohort	A Tb hospital	NALC-NaOH	AR	2%Ogawa	Loopamp MTBC	Sp	223/320	Yes
Moon 24	Korea (A)	Cohort	A university hospital	2%NALC-NaOH	ZN, AR	2%Ogawa	In-house (hspX)	Sp	35/303	Yes
Nimesh^25^	India (C)	rCohort, #	A hospital				In-house (sdaA)	Sp	18/236	Yes
Ou^26^	China (B)	Cohort	Microscopy centers		ZN	LJ	Loopamp MTBC	Sp	375/1329	Yes
Poudel^27^	Nepal (D)	Case-control	A Tb center	2%NALC-NaOH	AR	2%Ogawa	In-house (16S rRNA)	Sp	100/202	No
Rafati^28^	Iran (B)	#					In-house (16S rDNR)	Sp	10/50	Yes
Saito^29^	Japan (A)	Cohort, CR					Loopamp MTBC	Sp	25/161	Yes
Sethi^30^	India (C)	Cohort	A chest clinic	NALC-NaOH	ZN	LJ, MGIT	In-house (16s rRNA)	Sp	78/103	Yes
Thiong’o^31^	Kenya (C)	Thesis		NaOH		LJ	In-house (IS6110)	Sp	138/360	No
Watari^32^	Japan (A)	CR, Jap		NALC-NaOH			Loopamp MTBC	Sp	8/28	Yes

<Study> FIND: the Foundation for Innovative New diagnostics. <Income class> The World Bank income classification. A: high-income economy. B: upper-middle-income economy. C: lower-middle-income economy. D: low-income economy. <Design> pCohort: prospective cohort. rCohort: retrospective cohort. CR: conference report. Jpn: written in Japanese language. #: studies where only one sputum specimen was obtained per individual. These studies were used for analysis G (see Table 2, Fig. 3). <Decontamination> NALC-NaOH, N-acetyl-l-cysteine with sodium hydroxide; NaOH, sodium hydroxide. <Acid-fast stain>: ZN, Ziehl-Neelsen; AR, auramine-rhodamine. <Culture> MGIT, Mycobacteria Growth Indicator Tube; LJ, Löwenstein-Jensen. <LAMP assay> Loopamp MTBC: Loopamp MTBC Detection Kit targeting gyrB DNA and IS6110 DNA manufactured by EIKEN CHEMICAL CO., LTD. Eiken: Eiken Chemical Co., Ltd. (Tochigi, Japan), LAMP kit targeting gyrB DNA and IS6110 DNA. <Specimen> Sp: sputum, EP: extra-pulmonary specimen.

**Table 2:** Summary of results.

	(A)	(B)	(C)	(D)	(E)	(F)	(G)	(H)
Specimen	Sputum	Sputum	Sputum	Sputum	Sputum	Sputum	Sputum	Extra-pulmonary
Study quality	Any	High	Any	Any	Any	Any	Any	Any
LAMP assay	Any	Any	Loopamp MTBC	In-house	Loopamp MTBC	Loopamp MTBC	Any	Any
Smear	Any	Any	Any	Any	Positive	Negative	Any	Any
Studies	26	20	9	17	4	4	9#	1
Specimens	9330	5479	5283	4047	416	1460	4030	315
DOR, I^2^	145 (93–226), 19.8%	137 (76–247), 4.8%	126 (79–201), 8.1%	152 (72–321), 0%	66 (8.5–512), 9.0%	83 (48–144), 0%	130 (73–231), 0%	159 (36–712)
AUC	0.96 (0.950–0.98)	0.96 (0.95–0.98)	0.95 (0.93–0.97)	0.98 (0.98–0.99)	0.94 (0.81–1.00)	0.87 (0.84–0.91)	0.96 (0.94–0.98)	0.96
Sensitivity (%)	89.6 (85.6–92.6)	89.7 (85.0–93.1)	80.9 (76.0–85.1)	93.0 (88.9–95.7)	96.6 (90.4–98.8)	54.3 (34.7–72.6)	84.1 (78.9–88.2)	93.3 (77.9–99.2)
Specificity (%)	94.0 (91.0–96.1)	93.5 (88.9–96.3)	96.5 (94.7–97.7)	91.8 (86.4–95.1)	71.3 (37.1–91.3)	98.6 (97.3–99.1)	95.1 (92.6–96.8)	91.9 (88.1–94.8)
PLR	14.9 (9.8–22.8)	13.8 (8.0–24.2)	23.1 (15.1–35.2)	11.3 (6.9–19.2)	3.4 (1.5–11.1)	38.8 (19.3–72.5)	17.2 (11.3–36.3)	11.5 (7.1–17.5)
NLR	0.11 (0.08–0.15)	0.11 (0.07–0.16)	0.20 (0.15–0.25)	0.08 (0.05–0.12)	0.05 (0.02–0.16)	0.46 (0.28–0.66)	0.17 (0.12–0.22)	0.07 (0.01–0.33)

DOR: diagnostic odds ratio. AUC: area under hierarchical summary receiver operating characteristics curve. PLR: positive likelihood ratio. NLR: negative likelihood ratio. Brackets indicate 95% confidence interval.

^#^Studies that evaluated one specimen from one patient.
