# Supplementary material for: Diagnostic test accuracy of loop-mediated isothermal amplification assay for Mycobacterium tuberculosis: systematic review and meta-analysis
Source: Sci Rep. 2016 Dec 13;6:39090. doi: 10.1038/srep39090 (PMC5153623; doi:10.1038/srep39090)

**Diagnostic test accuracy of loop-mediated isothermal amplification assay for Mycobacterium tuberculosis: systematic review and meta-analysis**

**<<Supplement>>**

**Authors**

^1)^Kenjiro Nagai, ^1)^Nobuyuki Horita, ^1)^Masaki Yamamoto, ^1)^Toshinori Tsukahara, ^1)^Hideyuki Nagakura, ^1)^Ken Tashiro, ^1)^Yuji Shibata, ^1)^Hiroki Watanabe, ^1)^Kentaro Nakashima, ^1)^Ryota Ushio, ^1)^Misako Ikeda, ^1)^Atsuya Narita, ^1)^Akinori Kanai, ^1)^Takashi Sato, ^1)^Takeshi Kaneko.

^1)^Department of Pulmonology, Yokohama City University Graduate School of Medicine. 3-9, Fukuura, Kanazawa, Yokohama, Japan.

Supplementary Text 1. Search formulas.

EMBASE:

('tuberculosis'/exp OR tuberculosis OR 'tb'/exp OR tb OR mycobacterium OR 'mycobacterium'/exp) AND (lamp OR 'lamp'/exp OR 'loop-mediated isothermal amplification'/exp OR 'loop-mediated isothermal amplification') AND (sensitivity OR 'sensitivity'/exp OR specificity OR 'specificity'/exp OR 'predictive value'/exp OR 'predictive value' OR likelihood OR 'likelihood'/exp OR 'true positive' OR 'true negative' OR 'false positive' OR 'false negative').

Cochrane Library, title/abstract/keyword search without limitation:

(Tuberculosis OR TB OR mycobacteri*) AND (LAMP OR "loop-mediated isothermal amplification").

Web of Science Core collection advanced search without limitation:

TS=(Tuberculosis OR TB OR mycobacteri*) AND TS=(LAMP OR "loop-mediated isothermal amplification") AND TS=(sensitivity OR specificity OR "predictive value" OR likelihood OR "true positive" OR "true negative" OR "false positive" OR "false negative").

Supplementary Figure 1. QUADAS-2 risk of bias graph.


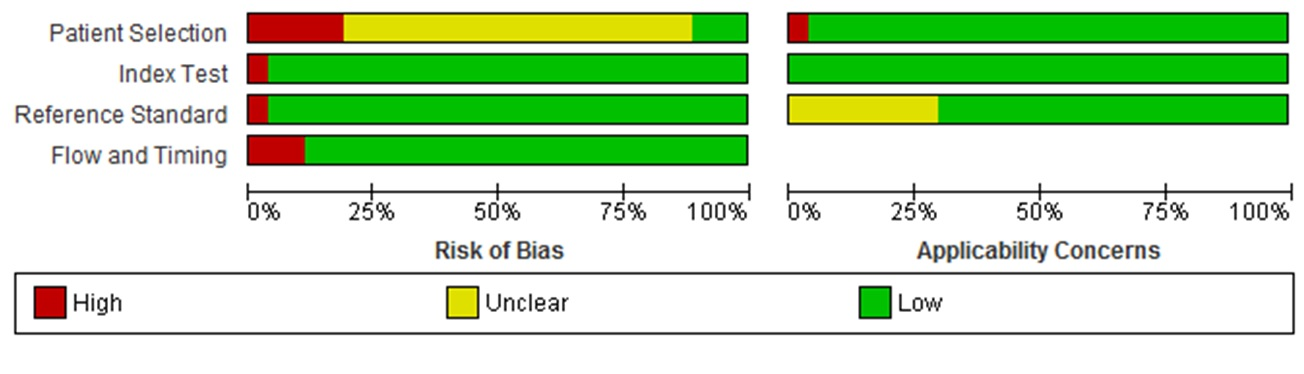


Supplementary Figure 2. QUADAS-2 risk of bias summary.


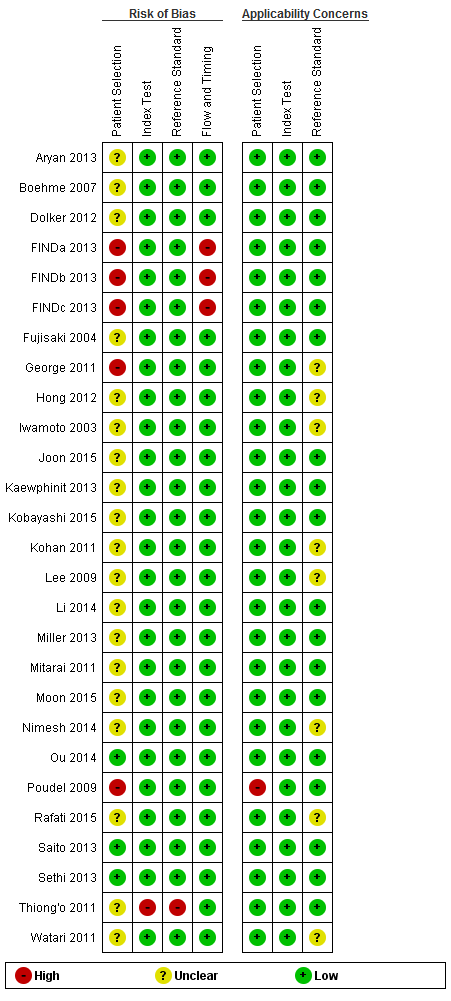

Supplement: Supplementary Information [file srep39090-s1.doc]
